# Effect of Drug-Coated Balloon in Side Branch Protection for *de novo* Coronary Bifurcation Lesions: A Systematic Review and Meta-Analysis

**DOI:** 10.3389/fcvm.2021.758560

**Published:** 2021-12-14

**Authors:** Yawei Zheng, Jie Li, Lingzhun Wang, Peng Yu, Haibo Shi, Lihua Wu, Jiandong Chen

**Affiliations:** ^1^Affiliated Hospital of Nanjing University of Chinese Medicine, Nanjing, China; ^2^Nanjing University of Chinese Medicine, Nanjing, China; ^3^Jiangsu Province Hospital of Chinese Medicine, Nanjing, China

**Keywords:** DCB, CBL, side branch, TLR, TLF, systematic review, meta-analysis

## Abstract

**Background:** At present, there are a variety of treatment strategies for percutaneous coronary intervention. The role of drug-coated balloon (DCB) in the treatment of side branch for *de novo* coronary bifurcated lesions (CBL) is unclear.

**Objective:** To examine the effect of DCB in side branch protection for *de novo* CBL.

**Methods:** Electronic databases, including Pubmed, Embase, the Web of science, Cochrance library, CNKI, CBM, WanFang Data and VIP were searched for studies that compared DCB with non-drug-coated balloon (NDCB) in side branch protection for *de novo* CBL from inception through July 7th, 2021. The primary outcome was target lesion revascularization (TLR). Secondary clinical outcomes included myocardial infarction (MI), cardiac death (CD). The angiographic outcomes included side branch late lumen loss (LLL), minimum lumen diameter (MLD), diameter stenosis (DS) and binary restenosis (BR). The target lesion failure (TLF) was also analyzed.

**Results:** A total of 10 studies, including 5 randomized controlled trials and 5 non-randomized observational studies, with 934 patients were included. Meta-analysis results of angiographic outcomes suggested that DCB group had the less LLL, DS and BR and the higher MLD compared with NDCB group at follow-up (*P* < 0.05). Meta-analysis results of clinical outcomes suggested that the significant difference in the TLR, MI and CD between DCB group and NDCB group has not been found yet (*P* > 0.05). However, the MACE of DCB group was significantly less than that of NDCB group at 9-month follow-up [OR = 0.21, 95%CI (0.05, 0.84), *P* = 0.03] and 12-month follow-up [OR = 0.45, 95%CI (0.22, 0.90), *P* = 0.02]. In addition, there was no significant difference in TLF between DCB group and NDCB group (*P* > 0.05).

**Conclusions:** DCB had great effect in side branch protection for *de novo* CBL at short and medium-term follow-up with no reduction in the procedural success rate.

**Systematic Review Registration:** https://www.crd.york.ac.uk/PROSPERO/display_record.php?RecordID=267426, PROSPERO [Identifier: CRD42021267426].

## Highlights

- DCB did not reduce the procedural success rate.
- DCB had great effect in side branch protection.
- DCB reduced the major adverse cardiac events.

## Introduction

Coronary bifurcation lesions (CBL) account for 15–20% of all percutaneous coronary intervention (PCI) and remain one of the most challenging lesions in interventional cardiology (1). Compared with coronary artery disease without bifurcation lesions, interventional treatment of CBL is not only more difficult in technology and more complicated in operation, but also poor in prognosis (2, 3). The optimal management, which can improve the procedural success rate and reduce long-term cardiac events, is still the subject of considerable debate. The provisional stenting strategy is currently considered the standard approach for the treatment of the majority of CBL (3, 4). The side branch may (or may not) be treated after the main vessel stent implantation according to the side branch flow and angiographic results. The advantage of the provisional stenting strategy is that the side branch treatment remains an open choice throughout the procedure. Early definite stent thrombosis is reduced when a single-stent strategy is used in CBL compared with the double-stent strategy (5). PCI using a provisional stenting strategy in CBL is associated with a reduction in all-cause mortality at long-term follow-up (6). Nevertheless, the side branch which has obvious functional value to patients cannot be lost during PCI. Long-term clinical outcomes are not only determined by the main vessel status after stent implantation, but also related to the side branch treatment. Therefore, it is a valuable problem that how to deal with the side branch. Drug-Coated Balloon (DCB), a combination of common balloon angioplasty and drug-eluting technology, releases antiproliferative drugs to the coronary artery wall locally, so as to inhibit intimal hyperplasia. In *de novo* CBL, DCB use in the side branch is an attractive approach (7). A study including 349 patients compared the side branch result using DCB vs. common balloon angioplasty indicates that DCB can reduce the side branch late lumen loss, but cannot reduce the side branch binary restenosis significantly at 9 months (8). However, the results are inconclusive, with many unanswered questions including actual impact on meaningful clinical endpoints. We performed a systematic review and meta-analysis to examine the effect of DCB in side branch protection for *de novo* CBL.

## Methods

The study protocol was registered with PROSPERO (CRD42021267426) and performed based on the PRISMA (Preferred Reporting Items for Systematic reviews and Meta-analyses) guidelines (9).

### Eligibility Criteria

Clinical studies comparing DCB with non-drug-coated balloon (NDCB) for the treatment of the side branch in *de novo* CBL were included. The side branch was treated with DCB in the treatment group, while in the control group, the side branch was treated with NDCB. In both groups, the side branch did not consider stent implantation. The type of study design included randomized controlled trial (RCT) and non-randomized observational study (nROS). Studies with incomplete data and no access to key data were excluded.

### Outcomes and Definitions

The primary outcome was target lesion revascularization (TLR). Secondary clinical outcomes included myocardial infarction (MI), cardiac death (CD). The major adverse cardiac events (MACE) which was defined as the sum of TLR, MI and CD was also analyzed. The angiographic outcomes included the side branch late lumen loss (LLL), minimum lumen diameter (MLD), diameter stenosis (DS) and binary restenosis (BR). The LLL was defined as the difference between the MLD measured post-procedure and the MLD measured at angiographic follow-up. The BR was defined as a diameter stenosis of at least 50%. The target lesion failure (TLF) was also concerned. The TLF was defined as the failure of side branch protection during operation, including complications such as dissection and thrombosis, and thrombolysis in myocardial infarction (TIMI) less than grade 3, or even salvage stent implantation.

### Search Strategy

Electronic databases, including Pubmed, Embase, the Web of science, Cochrance library, China National Knowledge Infrastructure (CNKI), China Biomedical database (CBM), Wanfang Data knowledge service platform (WanFang Data), and VIP information resource integration service platform (VIP) were searched without language restriction from inception through July 7th, 2021. The searched strategy was as follows: (“coronary bifurcation lesions” OR “bifurcation lesions” OR “CBL”) AND (“drug eluting balloon” OR “drug coated balloon” OR “drug balloon” OR “DEB” OR “DCB”).

### Study Screening and Data Extraction

Two researchers combined the eligibility criteria, independently screened the articles, extracted the data and cross-checked, and the differences were decided through discussion or arbitrated by the third researcher. Firstly, duplicate records were excluded through document management software. Then, the titles and abstracts of the remaining articles were read and the articles that obviously did not meet the eligibility criteria were excluded. Finally, after reading the full text of the remaining articles, the articles that meet the eligibility criteria were retained. Data were extracted from the included articles, including general information, methodological information, research object information, intervention information, and treatment outcome.

### Quality Assessment

The quality of each study was assessed by evaluating specific elements of each study design, with Jadad scales (10) and Newcastle-Ottawa Scales (NOS) (11) for RCTs and nROSs, respectively. In addition, the risk of bias for RCTs was assessed according to the Risk of Bias assessment Tool that was recommended by the Cochrane Collaboration (12).

### Statistical Analysis

Statistical analysis was performed using RevMan 5.3 and Stata 14 software. Continuous variables were expressed as mean difference (MD) expressed by 95% confidence intervals (CI). Binary variables were expressed as odds ratio (OR) expressed by 95%CI. First, clinical heterogeneity and methodological heterogeneity was assessed. Then, statistical heterogeneity was assessed using the Cochrane *Q* and *I*^2^ statistics (13). A *P* < 0.05 or *I*^2^ ≥ 50% suggested a high degree of statistical heterogeneity. The fixed-effect model was used when the heterogeneity was not significant, otherwise, the random-effect model was used (14). Inverse Variance pooling model was adopted in both the fixed-effects model and random-effects model. The trial sequential analysis was carried out to evaluate the reliability of the primary outcome results. Funnel plots were drawn to evaluate the possibility of publication bias when the number of studies was ≥10. The funnel plot of asymmetric distribution indicated that there was a high possibility of publication bias. In addition, the possibility of publication bias was analyzed by Egger's test. A *P* ≥ 0.05 indicated that the possibility of publication bias was less. Finally, in order to evaluate the stability of the results, we carried out sensitivity analysis by eliminating included studies item by item.

## Results

### Search Results and Study Characteristics

**Figure 1:**
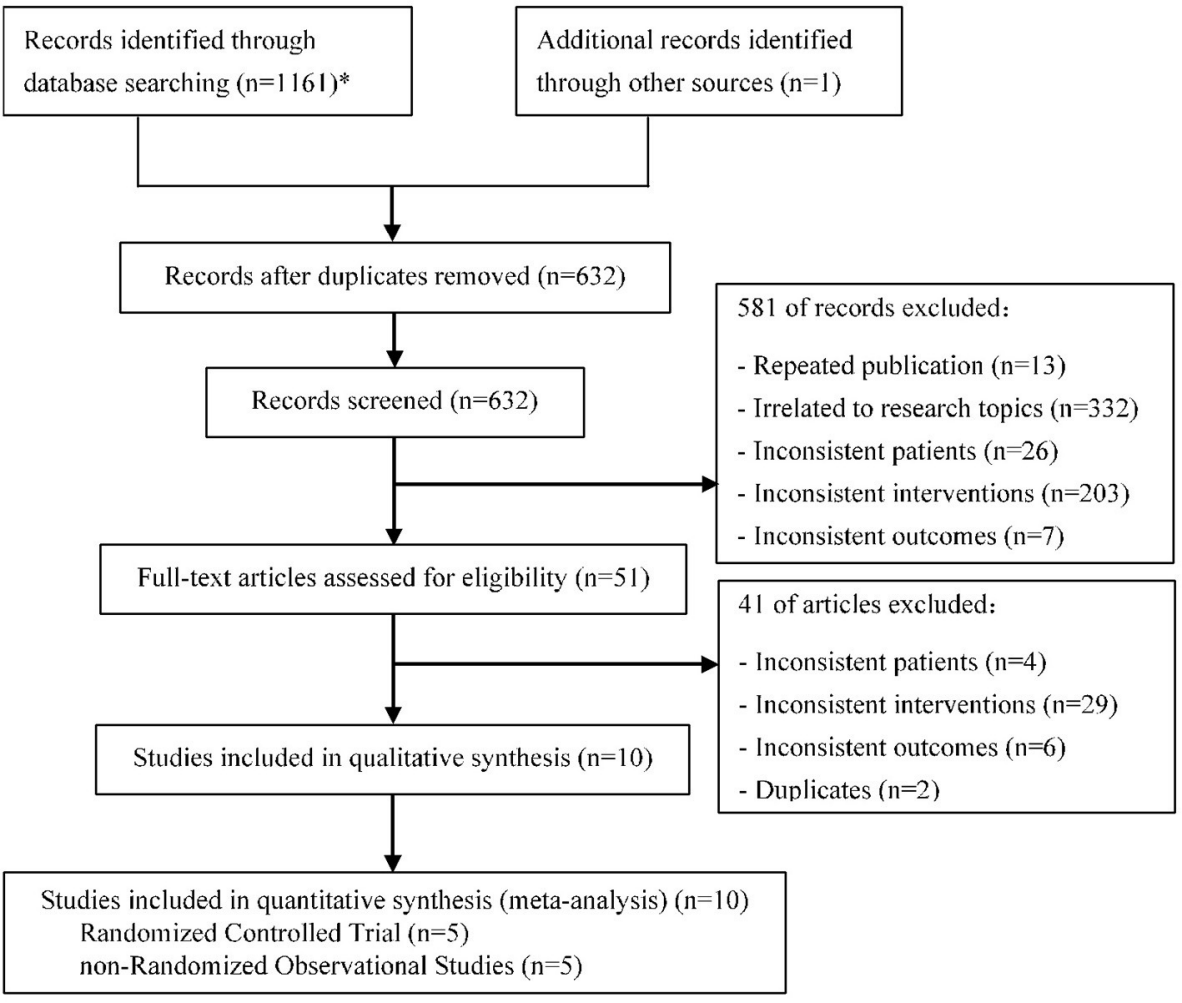
Flow chart for literature screening (PRISMA Flow Diagram). *PubMed (178); Embase (123); Web of science (313); Cochrane library (164); CNKI (174); CBM (61); WanFang (86); VIP (62).

After screening 1,162 initial articles using the electronic databases, 10 clinical studies (15–24) were finally identified, including 5 RCTs and 5 nROSs. The flow chart for literature screening was shown in Figure 1. Two of the 10 studies were multi-center studies (17, 18). The lesion location of 7 studies included the left main coronary artery (15, 16, 19, 20, 22–24), while the other 3 studies did not (17, 18, 21). There was no significant difference in age, gender, and risk factor (such as hypertension, diabetes mellitus, smoking status, et al.) between the treatment group and the control group in each study. In 9 studies, the main vessel was treated with stenting (15–17, 19–24), among which 2 studies did not specify the types of stents (19, 20). In all studies, the side branch was treated with DCB in the treatment group, while in the control group, the side branch was treated with NDCB. Eight of the 10 studies used the DCB of paclitaxel (16–18, 20–24), while the other two studies did not describe the specific type of DCB (15, 19). One study did not mention the presence or absence of pre-dilation (16), and the others used pre-dilation technology. In addition, all patients were treated with dual antiplatelet therapy. The longest follow-up time was 12 months. Characteristics of included studies were shown in Table 1.

**Table 1:** The baseline characteristics of included studies.

**References**	**Year**	**Design**	**Multi-center**	**Lesion location**	**CBL type**	**Pre-dilation**	**Gender (M/F)**		**Age (year)**		**Main vessel**	**Side branch**		**DCB type**	**Outcomes and follow-up**		**TLF report**	**Jadad/NOS**
							***T***	***C***	***T***	***C***		***T***	***C***		**Angiographic**	**Clinical**		
Bu et al. (15)	2021	RCT	N	Ang type	Lefevre I	Y	23/7	21/9	61.5 ± 7.3	59.1 ± 10.7	DES	DCB	NDCB	NA	TLR; MI; CD (12-months)	MLD; DS (6-months)	N	3^*^
Herrador et al. (16)	2013	nROS	N	Ang type	Ang type	NA	43/7	40/10	63.1 ± 11	61.9 ± 10.8	DES	DCB	NDCB	SeQuent Please	TLR; MI; CD (12-months)	LLL; MLD; DS; BR (12-months)	Y	8^Δ^
Jing et al. (17)	2020	RCT	Y	Non-LM	Medina (1,1,1); (0,1,1); (1,0,1)	Y	90/23	71/38	59.9 ± 10.1	61.8 ± 9.4	DES	DCB	NDCB	Bingo	TLR; MI; CD (1/6/9-months)	LLL; MLD; DS (9-months)	Y	6^*^
Kleber et al. (18)	2016	RCT	Y	LAD; LCX; RCA	Medina (0,0,1); (0,1,0); (0,1,1)	Y	24/8	23/9	66 ± 12	69 ± 10	no-stenting	DCB	NDCB	SeQuent Please	TLR; MI; CD (9-months)	LLL; MLD; DS; BR (9-months)	Y	6^*^
Li et al. (19)	2019	nROS	N	LM	Medina (1,1,1)	Y	27/17	37/29	58.8 ± 10.2	58.3 ± 9.5	any stent	DCB	NDCB	NA	TLR; MI; CD (12-months)	DS (12-months)	N	8^Δ^
Xia et al. (20)	2019	nROS	N	LM; LAD; LCX	Medina (1,1,1); (0,1,1); (1,0,1)	Y	40/9	42/24	61.14 ± 10.74	58.46 ± 11.87	any stent	DCB	NDCB	SeQuent Please	MI; CD (6/9/12-months)	—	Y	9^Δ^
Zhang (21)	2019	nROS	N	LAD; LCX; RCA	Ang type	Y	25/21	27/28	64.46 ± 4.14	65.02 ± 5.08	DES	DCB	NDCB	SeQuent Please	MI; CD (3/6/12-months)	—	Y	8^Δ^
Zhang et al. (22)	2019	nROS	N	Ang type	Medina (1,1,1); (0,1,1); (1,0,1)	Y	21/7	22/10	62.0 ± 8.3	58.5 ± 10.8	DES	DCB	NDCB	SeQuent Please	TLR; MI; CD (9-months)	LLL; MLD (9-months)	Y	7^Δ^
Zhao (23)	2017	RCT	N	Ang type	Medina (1,1,1); (0,1,1); (1,0,1)	Y	23/6	25/6	57.5 ± 11.6	61.2 ± 9.2	DES	DCB	NDCB	SeQuent Please	TLR; MI; CD (12-months)	LLL; MLD; BR (9-months)	Y	3^*^
Zong et al. (24)	2018	RCT	N	Ang type	Medina (1,1,1); (0,1,1); (1,0,1)	Y	13/8	11/10	57.5 ± 7.4	55.2 ± 7.3	DES	DCB	NDCB	SeQuent Please	TLR; MI; CD (6-months)	LLL; MLD (6-months)	N	4^*^

* *Jadad*.

Δ *NOS*.

*CBL, coronary bifurcation lesions; M, male; F, female; T, treatment group; C, control group; NOS, Newcastle-Ottawa Scales; RCT, randomized controlled trial; nROS, non-randomized observational study; LM, Left main coronary artery; LAD, Left anterior descending artery; LCX, Left circumflex artery; RCA, Right coronary artery; DES, drug-eluting stent; DCB, drug-coated balloon; NDCB, non-drug-coated balloon; TLF, Target lesion failure; TLR, Target lesion revascularization; MI, Myocardial infarction; CD, Cardiac death; LLL, Late lumen loss; MLD, Minimum lumen diameter; DS, Diameter stenosis; BR, Binary restenosis; NA, unavailable*.

### Risk of Bias in the Included Studies

**Figure 2:**
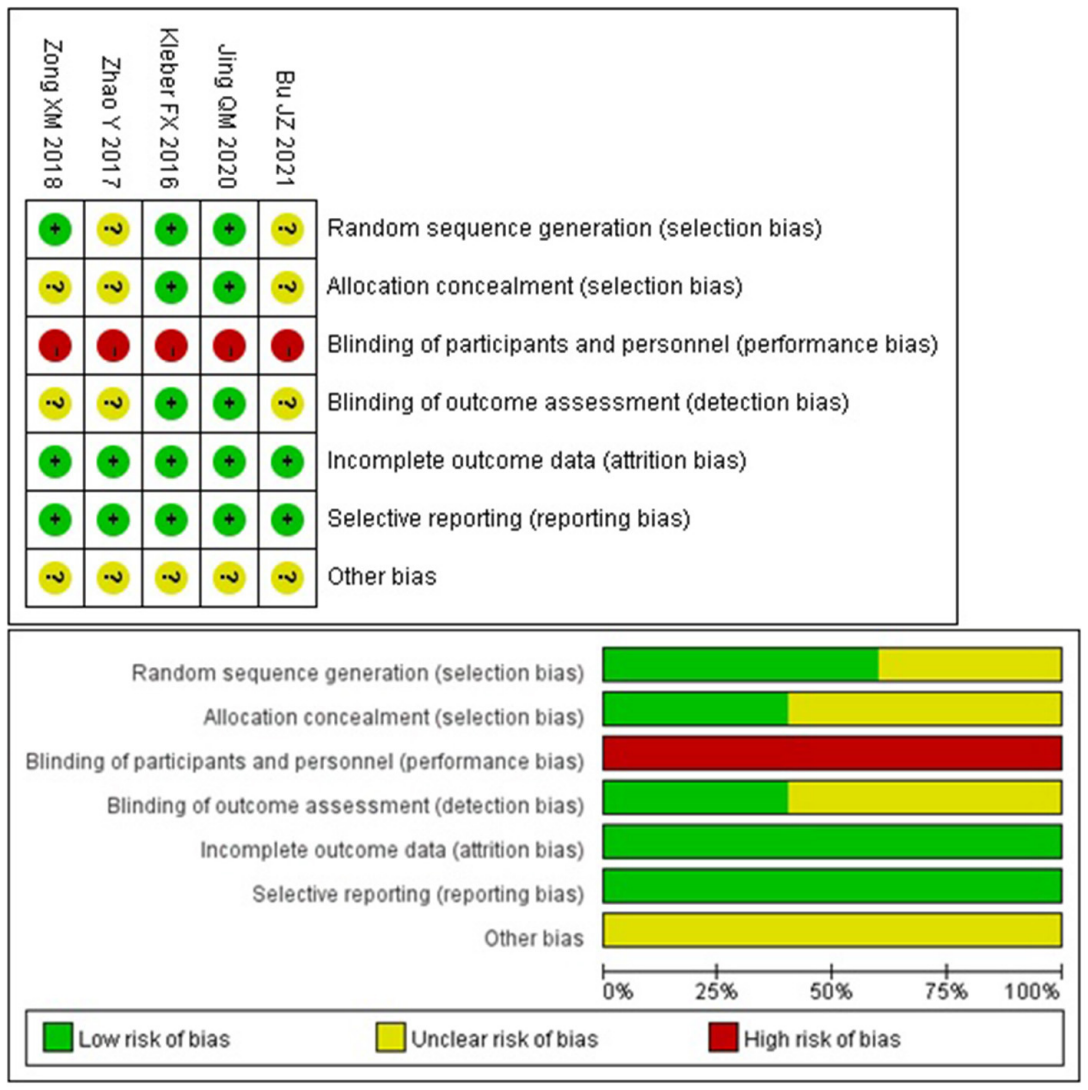
Risk of bias for RCTs (Cochrane risk tool).

The quality of each study was assessed by evaluating specific elements of each study design, with Jadad or NOS for RCTs and nROSs, respectively. The studies included were of relatively high quality (Table 1). In addition, we assessed the risk of bias for RCTs according to the Cochrane Collaboration Tool. The risk of bias in the included studies was relatively low (Figure 2). Only two studies were multicenter design (17, 18). Three studies explained the specific method of random allocation (17, 18, 24), and two studies only mentioned “randomization” (15, 23).

### Target Lesion Revascularization

**Figure 3:**
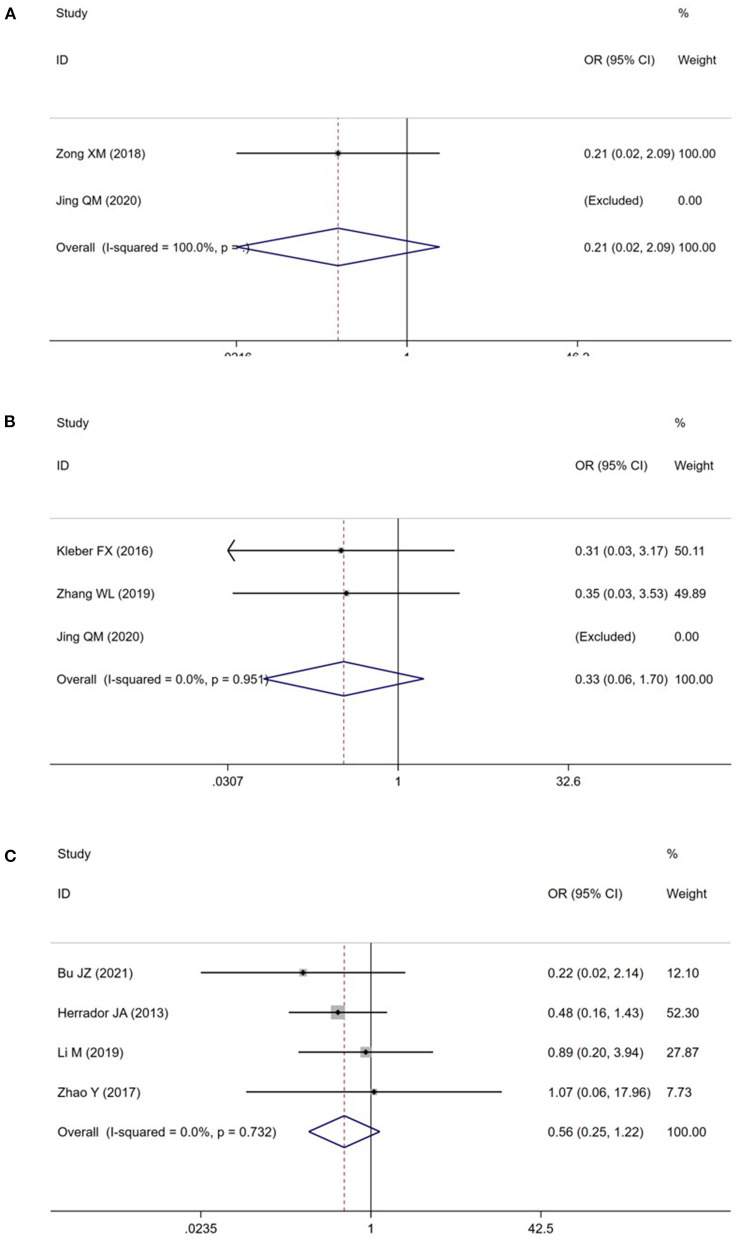
Meta analysis for the target lesion revascularization (**A**: at 6-month follow-up; **B**: at 9-month follow-up; **C**: at 12-month follow-up).

**Figure 4:**
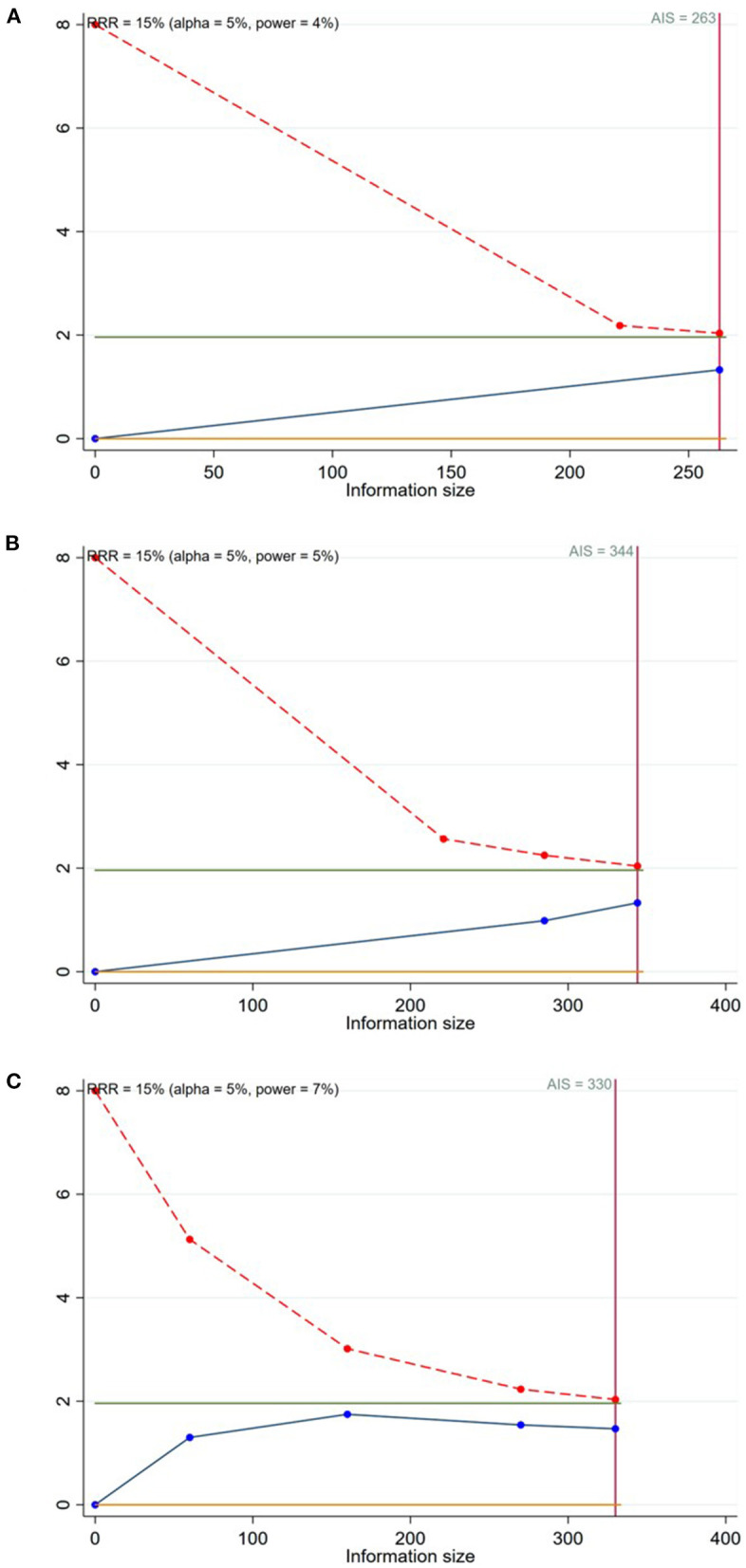
Trial sequential analysis for the target lesion revascularization (**A**: at 6-month follow-up; **B**: at 9-month follow-up; **C**: at 12-month follow-up).

A total of 8 studies (15–19, 22–24) reported the TLR in patients with CBL (Figures 3A–C). Meta-analysis results suggested that there was no significant difference in the TLR between DCB group and NDCB group at 6-month follow-up [OR = 0.21, 95%CI (0.02, 2.09), *P* = 0.18], 9-month follow-up [OR = 0.33, 95%CI (0.06, 1.70), *P* = 0.18] and 12-month follow-up [OR = 0.56, 95%CI (0.25, 1.22), *P* = 0.14] (Supplementary Figure S1). We applied Egger's test to evaluate publication bias. Although the difference was not statistically significant, we found a trend of DCB group with significant advantages. Trial sequential analysis was performed to evaluate the reliability of the results (Figures 4A–C). The statistical power was only 4, 5, and 7%, respectively, which indicated that the results of TLR lacked reliability due to insufficient sample size. A *p* (*P* = 0.949) value more than 0.05 was considered to be unlikely to exist publication bias.

### Secondary Clinical Outcomes

**Figure 5:**
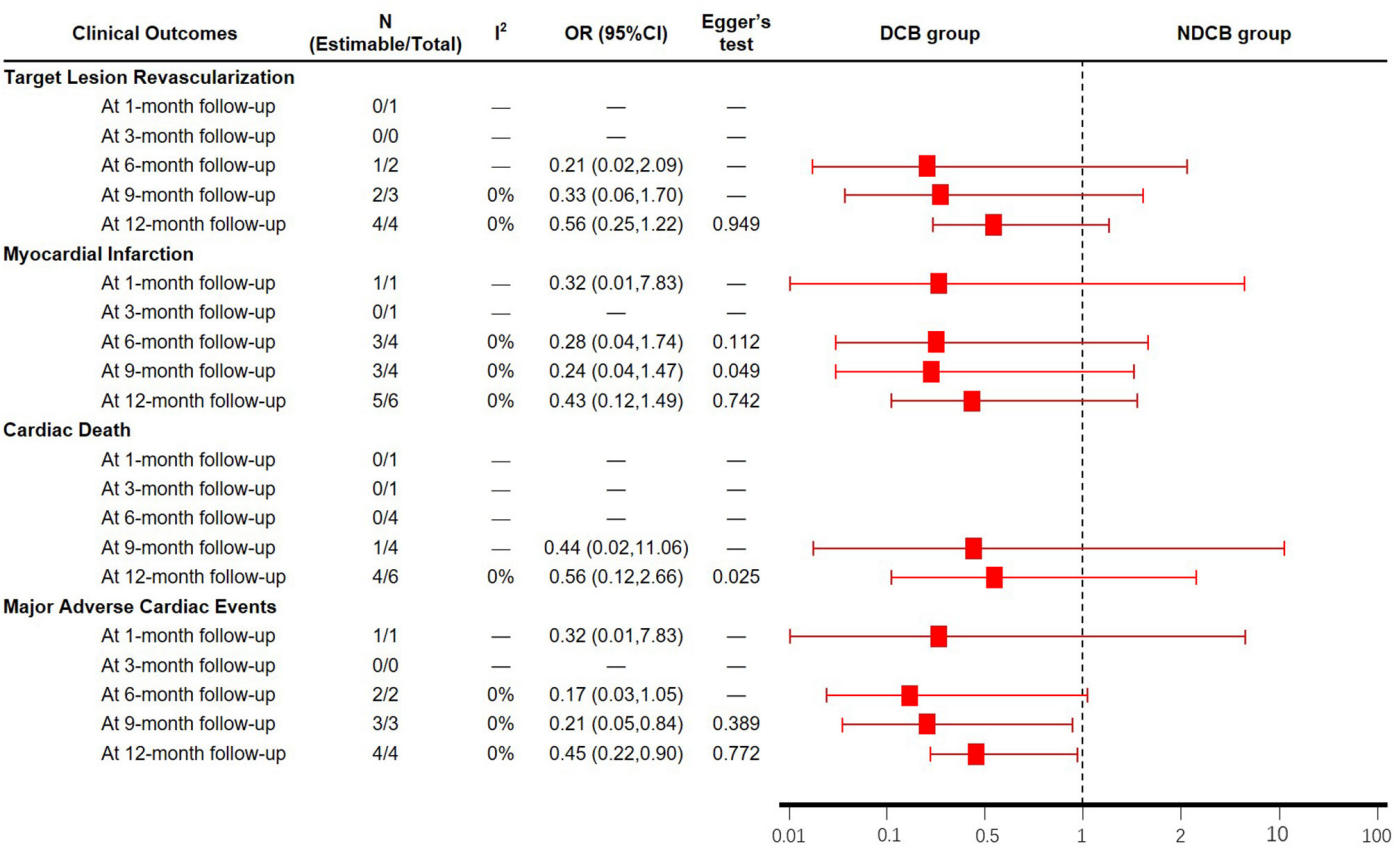
Meta-analysis results of the clinical outcomes.

A total of 10 studies (15–24) reported the MI and CD in patients (Figure 5). Meta-analysis results suggested that there was no significant difference in the MI and CD between DCB group and NDCB group at follow-up (*P* > 0.05) (Supplementary Figures S2, S3). Egger's test results suggested that there was great possibility of publication bias in MI at 9-month follow-up (*P* = 0.049) and CD at 12-month follow-up (*P* = 0.025). The MACE was defined as the sum of TLR, MI and CD. A total of 8 studies (15–19, 22–24) reported the TLR, MI and CD at the same time (Figure 5). Meta-analysis results suggested that the MACE of DCB group was significantly less than that of NDCB group at 9-month follow-up [OR = 0.21, 95%CI (0.05, 0.84), *P* = 0.03] and 12-month follow-up [OR = 0.45, 95%CI (0.22, 0.90), *P* = 0.02] (Supplementary Figure S4). However, there was no significant difference in the MACE between DCB group and NDCB group at 1-month follow-up and 6-month follow-up (*P* > 0.05). Egger's test results suggested that there was less possibility of publication bias (*P* > 0.05).

### Angiographic Outcomes

**Figure 6:**
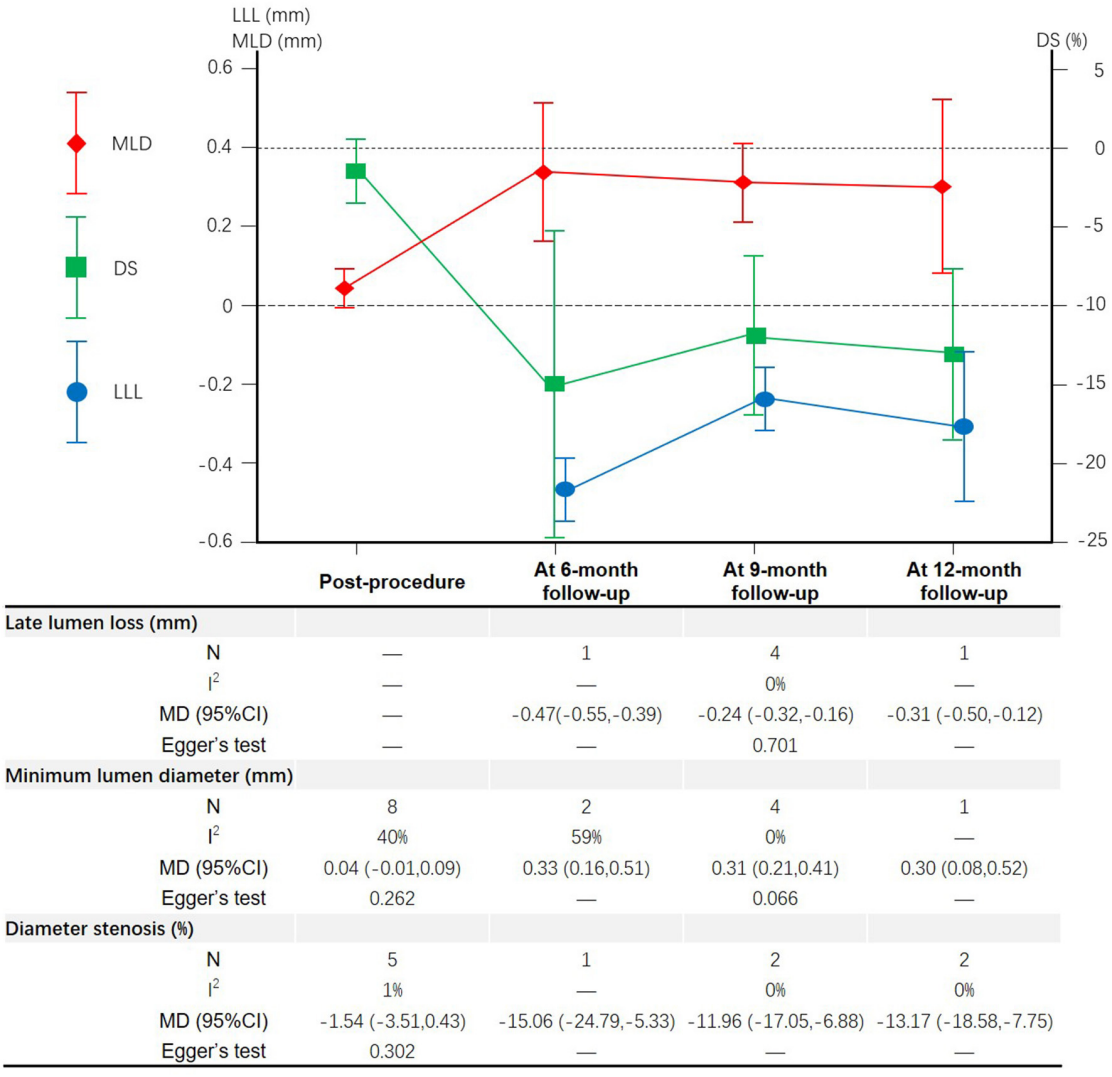
Meta-analysis results of the late lumen loss, minimum lumen diameter and diameter stenosis.

A total of 8 studies (15–18, 21–24) and 5 studies (15–19) reported the MLD and DS measured post-procedure in patients with CBL, respectively (Figure 6). Meta-analysis results suggested that there was no significant difference in the MLD and DS measured post-procedure between DCB group and NDCB group (*P* > 0.05) (Supplementary Figures S5, S6). A total of 6 studies (16–18, 22–24), 7 studies (15–18, 22–24), and 5 studies (15–19) reported the LLL, MLD, and DS measured at follow-up, respectively (Figure 6). The LLL of DCB group was significantly less than that of NDCB group at 6-month follow-up [MD = -0.47, 95%CI (-0.55, -0.39), *P* < 0.00001], 9-month follow-up [MD = -0.24, 95%CI (-0.32, -0.16), *P* < 0.00001] and 12-month follow-up [MD = -0.31, 95%CI (-0.50, -0.12), *P* = 0.002] (Supplementary Figure S7). The MLD of DCB group was significantly more than that of NDCB group at 6-month follow-up [MD = 0.33, 95%CI (0.16, 0.51), *P* = 0.0002], 9-month follow-up [MD = 0.31, 95%CI (0.21, 0.41), *P* < 0.00001] and 12-month follow-up [MD = 0.30, 95%CI (0.08, 0.52), *P* = 0.006] (Supplementary Figure S5). The DS of DCB group was significantly less than that of NDCB group at 6-month follow-up [MD = -15.06, 95%CI (-24.79, -5.33), *P* = 0.002], 9-month follow-up [MD = -11.96, 95%CI (-17.05, -6.88), *P* < 0.00001] and 12-month follow-up [MD = -13.17, 95%CI (-18.58, -7.75), *P* < 0.00001] (Supplementary Figure S6). Egger's test results suggest that there was less possibility of publication bias (*P* > 0.05).

**Figure 7:**
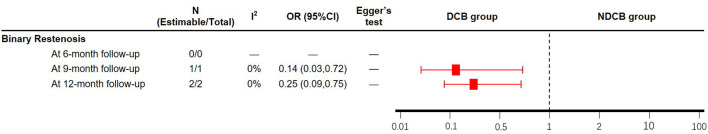
Meta-analysis results of the binary restenosis.

A total of 3 studies (16, 18, 23) reported the BR measured at follow-up (Figure 7). The BR of DCB group was significantly less than that of NDCB group at 9-month follow-up [OR = 0.14, 95%CI (0.03, 0.72), *P* = 0.02] and 12-month follow-up [OR = 0.25, 95%CI (0.09, 0.75), *P* = 0.01] (Supplementary Figure S8). The number of studies was too small to apply Egger's test.

### Target Lesion Failure

**Figure 8:**
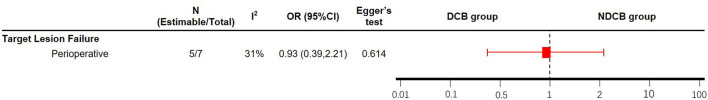
Meta-analysis results of the target lesion failure.

A total of 7 studies (16–18, 20–23) reported the TLF (Figure 8). Meta-analysis results suggested that there was no significant difference in the TLF between DCB group and NDCB group [OR = 0.93, 95%CI (0.39, 2.21), *P* = 0.86] (Supplementary Figure S9). Egger's test results suggest that there was less possibility of publication bias (*P* = 0.614).

### Sensitivity Analysis

**Figure 9:**
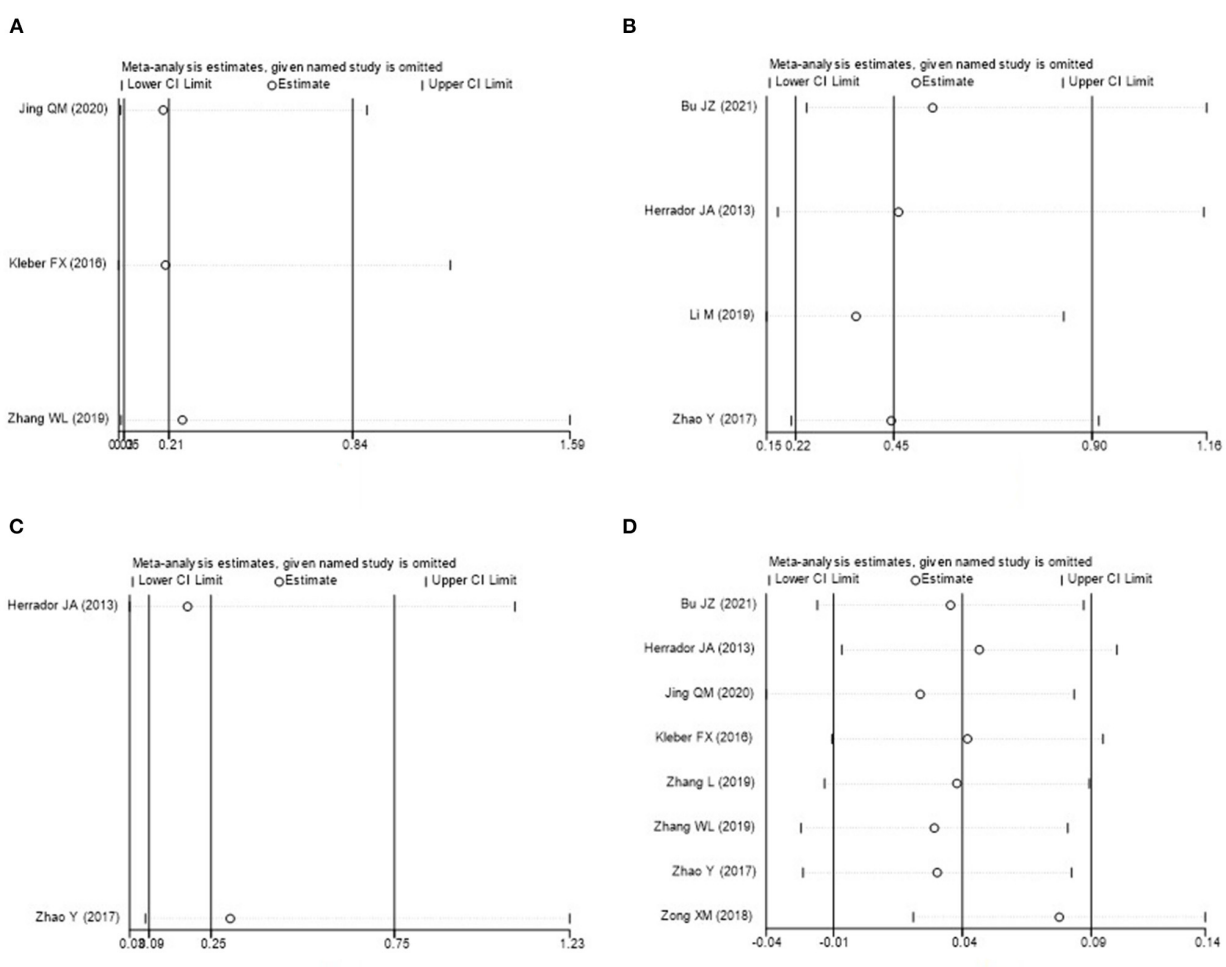
Sensitivity analysis (**A**: MACE at 9-month follow-up; **B**: MACE at 12-month follow-up; **C**: BR at 12-month follow-up; **D**: MLD measured post-procedure).

Sensitivity analysis was carried out though seriatim excluding one trial each time and re-performing meta-analysis of the remaining trials. When Kleber FX's or Zhang WL's article was eliminated, the difference of MACE between DCB group and NDCB group at 9-month follow-up became no significant (*P* = 0.07 or *P* = 0.14). When Bu JZ's or Herrador JA's article was eliminated, the difference of MACE between DCB group and NDCB group at 12-month follow-up became no significant (*P* = 0.11 or *P* = 0.10). When Herrador JA's or Zhao Y's article was eliminated, the difference of BR between DCB group and NDCB group at 12-month follow-up became no significant (*P* = 0.06 or *P* = 0.09). These changes were thought to be caused by the decrease of sample size. When Zong XM's article was eliminated, the difference of MLD measured post-procedure between DCB group and NDCB group became significant [MD = 0.08, 95%CI (0.02, 0.14), *P* = 0.009] (Figure 9). However, this difference lacked clinical value. The other results and statistical heterogeneity did not change significantly when eliminating included studies item by item, which indicated that the results were stable.

## Discussion

A CBL was a lesion occurring at, or adjacent to, a significant division of a major coronary artery (25). The long-term clinical outcomes of CBL patients mainly depended on the state of the main vessel after stent implantation. At the same time, the significant side branch that the operators do not want to lose after PCI should not be ignored. Bifurcation treatment techniques should be considered when the opening of the side branch may affect the prognosis or the stenosis of the side branch may cause symptoms. The provisional stenting strategy was currently considered the standard approach for the treatment of the majority of CBL. The advantage of balloon angioplasty instead of stent implantation in the side branch treatment was that it was associated with a reduction in definite stent thrombosis, all-cause mortality while restoring anatomy (5, 6, 26). However, the risk of binary restenosis in the long term was still high after the application of traditional balloon angioplasty in the side branch. With the continuous combination of drug-coated technology and traditional balloon angioplasty, DCB came into being. DCB was to carry the anti-intimal hyperplasia drug on the balloon surface by matrix coating or nano-microporous technology. When the DCB expanded, the drug it carried was released to the blood vessel wall, thus inhibiting intimal hyperplasia and reduce vascular endothelial inflammation and thrombosis (27).

DCB combined the advantages of common balloon angioplasty and drug-eluting stent implantation. Several single-arm trials suggested that the DCB angioplasty for the side branch with main vessel stenting seemed to improve the clinical outcome at short and medium-term follow-up (28–31). DCB had the advantage of the lack of foreign material in the artery and got rid of the high incidence of restenosis after stent implantation. In the 15th consensus document from the European Bifurcation Club, DCB technology was considered to as pivotal to enhance clinical outcomes (7). This study systematically evaluated the procedural success, cardiovascular events and side branch protection of DCB for *de novo* CBL. Besides, angiographic and clinical outcomes according to different follow-up nodes was considered.

In this systematic review and meta-analysis of 10 studies, including 5 RCTs and 5 nROSs of 934 patients with *de novo* CBL, we documented that DCB not only had great effect in reducing LLL, DS and BR, and increasing MLD of side branch for *de novo* CBL with no reduction in the procedural success rate, but also reduced the MACE.

In term of angiographic outcomes, meta-analysis results suggested that there was no significant difference in the MLD and DS measured post-procedure between DCB group and NDCB group. However, DCB group had lower LLL, DS and BR measured at follow-up and higher MLD measured at follow-up compared with NCB group. The biggest benefit occurred at 6-month follow-up. The results showed that the immediate effect of the DCB and NDCB in side branch protection was similar, but over time, the DCB gradually showed its advantages of the side branch protection. The side branch protection benefited from drug release.

In term of clinical outcomes, meta-analysis results suggested that the MACE of DCB group was significantly less than that of NDCB group at 9-month follow-up and 12-month follow-up. This result proved that the application of DCB in the side branch can improve the clinical outcomes of patients with CBL. However, due to the limitation of sample size, there was no significant difference in the MACE between the two groups at 1-month follow-up and 6-month follow-up. The difference in the TLR, MI and CD between DCB group and NDCB group was not significant in this study. As shown by trial sequential analysis results, the low incidence of TLR lead to the need for a larger sample size with enough statistical power to find the significant difference between groups. For MI and CD, the negative results may be caused by the same reason. Therefore, it may be not that there was no significant difference in TLR, MI and CD between the two groups, but that significant difference had not been found yet. More large-sample and high-quality RCTs need to be implemented to draw such a conclusion. According to current evidence, the reduction of MACE was not transparent enough to prove that the side branch protective effect of DCB was successfully transformed into the improvement of clinical outcomes.

In addition, there was no significant difference in TLF between DCB group and NDCB group. The procedural success rate of DCB and NDCB was similar. It was safe and reliable to apply DCB angioplasty to the side branch in the treatment of patients with CBL. In European Society of Cardiology guidelines, DCB was recommended for the treatment of in-stent restenosis within bare-metal stent or drug eluting stent while there were no convincing data to support the use of DCB angioplasty for *de novo* disease (3). This study systematically examined the effect of DCB in side branch protection for *de novo* CBL. However, there were still many unanswered questions including the appropriate lesion location selection (non-left main coronary artery or left main coronary artery), appropriate side branch selection (vessel diameter less or more than 2.8 mm), coating drugs selection (Paclitaxel, Zotarolimus or Sirolimus), and balloon angioplasty technique (DCB with or without final kissing ballooning or repeat POT).

## Limitation

However, there were several limitations in our study. First, only articles published in English and Chinese were incorporated, which led to a potential selection bias. Second, because of the lack of background data for studies in meta-analyses, the data were not further stratified by other factors that may affect outcomes. Third, there was no significant difference in the TLR between groups accompanied by poor statistical power. This result was not reliable due to the limitation of sample size. It's the same reason for MI and CD. At present, several trials are under study, which is expected to clarify this problem. Forth, sensitivity analysis suggested that several results of this study were not stable because the number of trials for each indicator was small, accompanied by a small sample size. Fifth, the follow-up time of the included trials was between 1 and 12 months, so as to obtain the conclusion of short and medium-term follow-up, while no long-term follow-up outcome could be evaluated.

## Conclusion

Current evidence indicated that DCB had great effect in side branch protection for *de novo* CBL at short and medium-term follow-up with no reduction in the procedural success rate. Due to the limitation of the quantity and quality of the included studies, the conclusions of this study still need to be confirmed by more high-quality, multi-center and large-sample size RCTs. The relevant systematic review should be updated in time when new trials are published.

## Data Availability Statement

The original contributions presented in the study are included in the article/Supplementary Material, further inquiries can be directed to the corresponding authors.

## Author Contributions

YZ and JC conceived and designed the experiments and revised the manuscript. YZ, JL, and LWa performed the experiments. YZ, JL, and LWu and analyzed the data. YZ, JL, PY, and HS wrote the manuscript. All authors reviewed and approved the manuscript prior to submission.

## Funding

This study was supported by grants from the National Natural Science Foundation of China (Grant No. 81973763).

## Conflict of Interest

The authors declare that the research was conducted in the absence of any commercial or financial relationships that could be construed as a potential conflict of interest.

## Publisher's Note

All claims expressed in this article are solely those of the authors and do not necessarily represent those of their affiliated organizations, or those of the publisher, the editors and the reviewers. Any product that may be evaluated in this article, or claim that may be made by its manufacturer, is not guaranteed or endorsed by the publisher.
