# Supplementary material for: Effect of Drug-Coated Balloon in Side Branch Protection for de novo Coronary Bifurcation Lesions: A Systematic Review and Meta-Analysis
Source: Front Cardiovasc Med. 2021 Dec 14;8:758560. doi: 10.3389/fcvm.2021.758560 (PMC8712469; doi:10.3389/fcvm.2021.758560)
Supplement: Supplementary file 1 [file Data_Sheet_1.docx]

**Supplementary Materials for**

**“Effect of Drug-Coated Balloon in side branch protection for de novo Coronary Bifurcation Lesions: A Systematic Review and Meta-analysis”**

**Authors:**

Yawei Zheng^1,3^; Jie Li^1,2,3^; Lingzhun Wang^1,2^; Peng Yu^1,2^; Haibo Shi^1,2^; Lihua Wu^1,2^; Jiandong Chen^1,2,3^

**Affiliation:**

^1^Aﬃliated Hospital of Nanjing University of Chinese Medicine, Nanjing, China.

^2^Jiangsu Province Hospital of Chinese Medicine, Nanjing, China.

^3^Nanjing University of Chinese Medicine, Nanjing, China.

^*^Correspondence: Jiandong Chen: chenjdcardiologist@njucm.edu.cn.

Jie Li: jellylee8803@126.com.

**Figure S1** Forest plots for the target lesion revascularization (A: at 1-month follow-up; B: at 6-month follow-up; C: at 9-month follow-up; D: at 12-month follow-up).

**A:** at 1-month follow-up

**
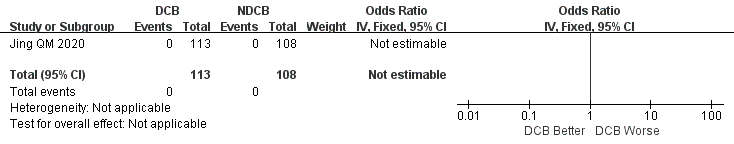
**

**B:** at 6-month follow-up

**
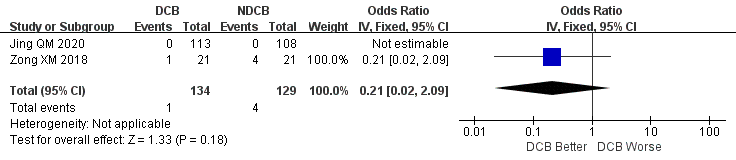
**

**C:** at 9-month follow-up

**
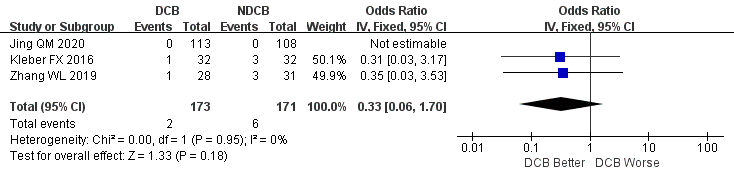
**

**D:** at 12-month follow-up

**
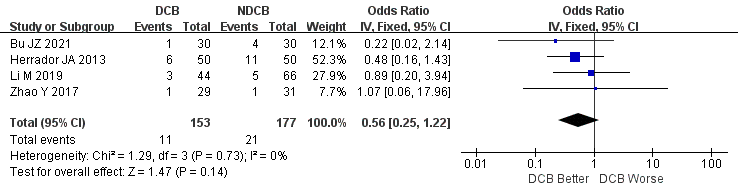
**

**Figure S2** Forest plots for the myocardial infarction (A: at 1-month follow-up; B: at 3-month follow-up; C: at 6-month follow-up; D: at 9-month follow-up; E: at 12-month follow-up).

**A:** at 1-month follow-up

**
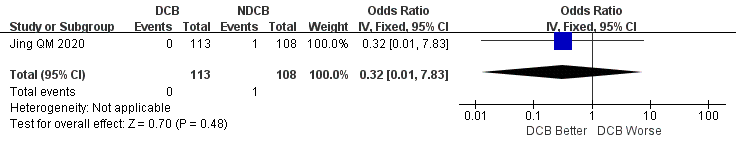
**

**B:** at 3-month follow-up

**
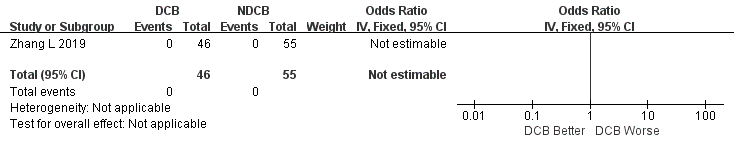
**

**C:** at 6-month follow-up

**
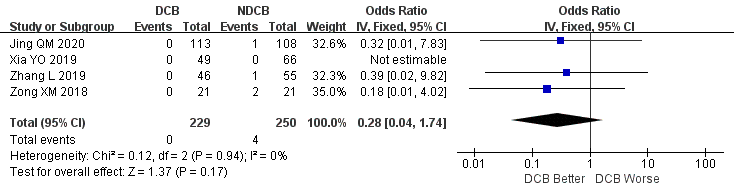
**

**D:** at 9-month follow-up

**
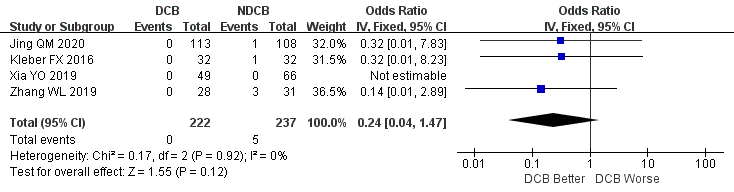
**

**E:** at 12-month follow-up

**
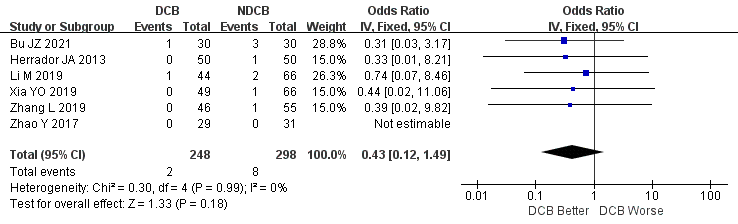
**

**Figure S3** Forest plots for the cardiac death (A: at 1-month follow-up; B: at 3-month follow-up; C: at 6-month follow-up; D: at 9-month follow-up; E: at 12-month follow-up).

**A:** at 1-month follow-up

**
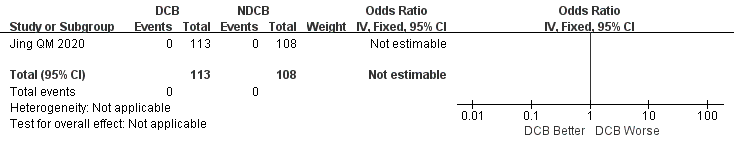
**

**B:** at 3-month follow-up

**
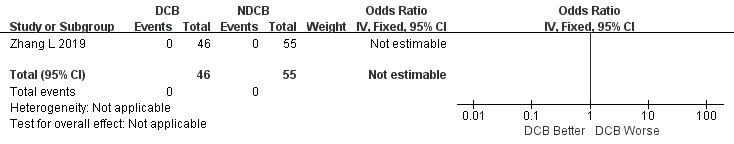
**

**C:** at 6-month follow-up

**
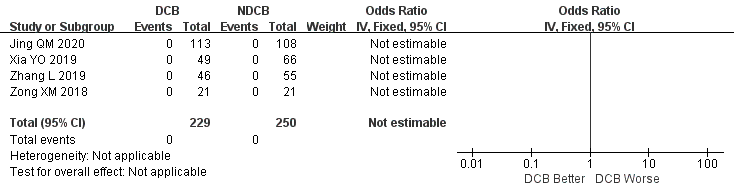
**

**D:** at 9-month follow-up

**
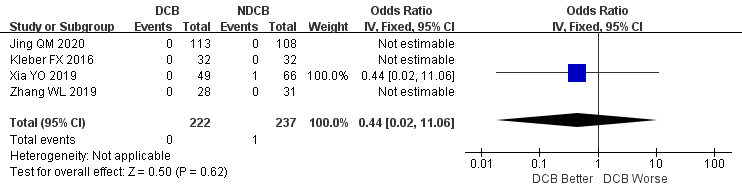
**

**E:** at 12-month follow-up

**
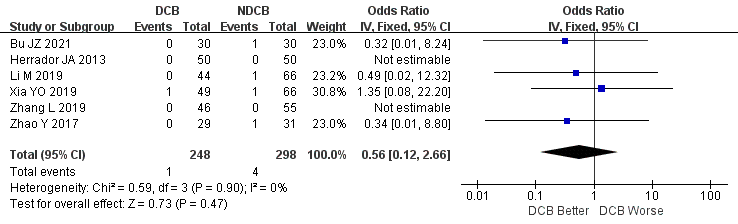
**

**Figure S4** Forest plots for the major adverse cardiac events (A: at 1-month follow-up; B: at 6-month follow-up; C: at 9-month follow-up; D: at 12-month follow-up).

**A:** at 1-month follow-up

**
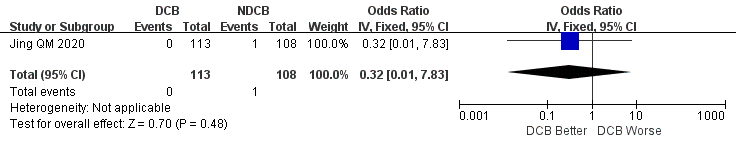
**

**B:** at 6-month follow-up

**
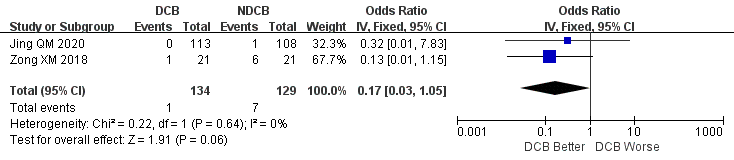
**

**C:** at 9-month follow-up

**
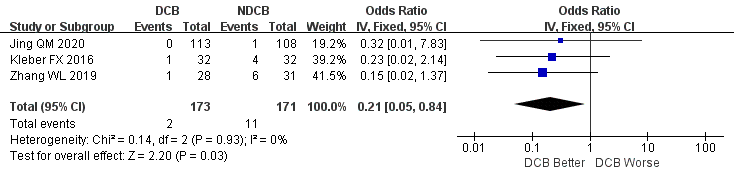
**

**D:** at 12-month follow-up

**
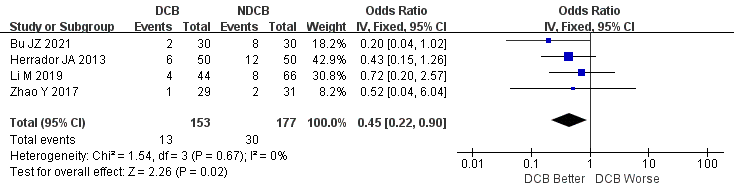
**

**Figure S5** Forest plots for the minimum lumen diameter (A: post-procedure; B: at 6-month follow-up; C: at 9-month follow-up; D: at 12-month follow-up).

**A:** post-procedure

**
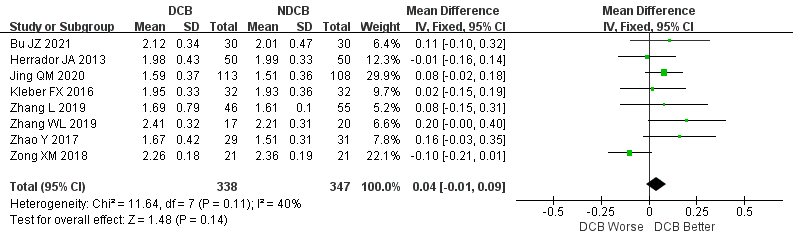
**

**B:** at 6-month follow-up

**
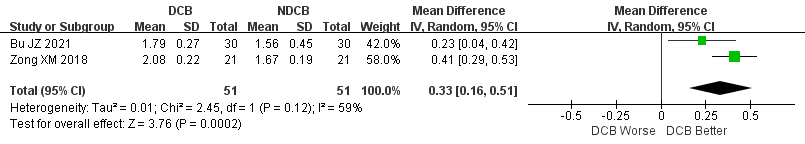
**

**C:** at 9-month follow-up

**
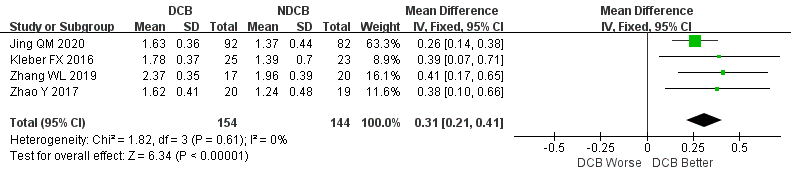
**

**D:** at 12-month follow-up

**
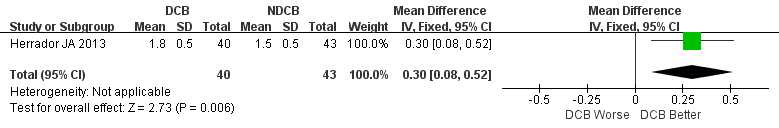
**

**Figure S6** Forest plots for the diameter stenosis (A: post-procedure; B: at 6-month follow-up; C: at 9-month follow-up; D: at 12-month follow-up).

**A:** post-procedure

**
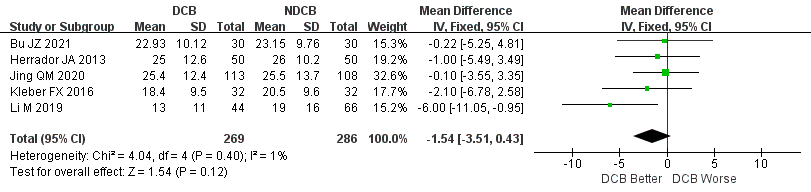
**

**B:** at 6-month follow-up

**
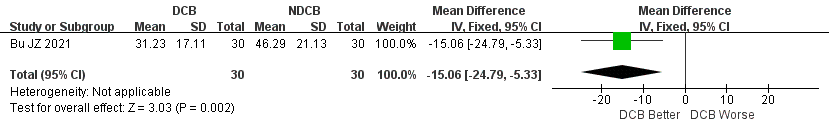
**

**C:** at 9-month follow-up

**
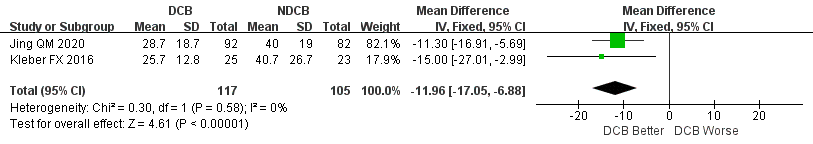
**

**D:** at 12-month follow-up

**
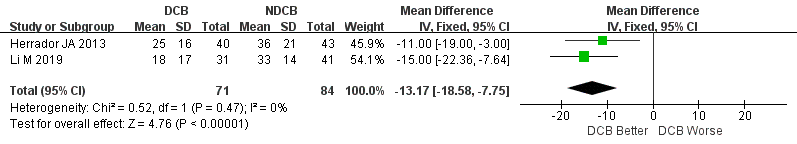
**

**Figure S7** Forest plots for the late lumen loss (A: at 6-month follow-up; B: at 9-month follow-up; C: at 12-month follow-up).

**A:** at 6-month follow-up

**
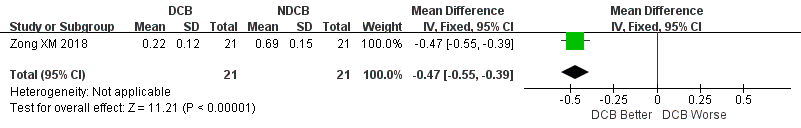
**

**B:** at 9-month follow-up

**
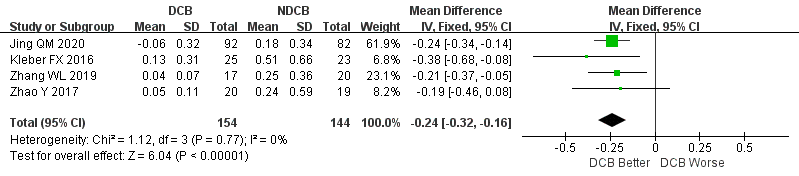
**

**C:** at 12-month follow-up

**
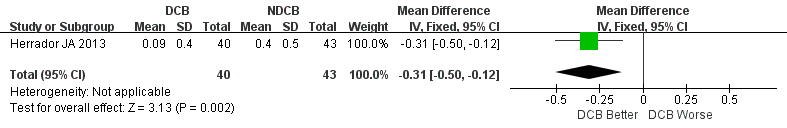
**

**Figure S8** Forest plots for the binary restenosis (A: at 9-month follow-up; B: at 12-month follow-up).

**A:** at 9-month follow-up

**
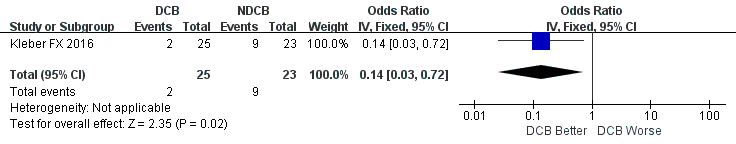
**

**B:** at 12-month follow-up

**
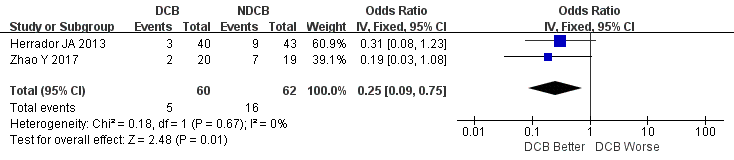
**

**Figure S9** Forest plots for the target lesion failure.

**
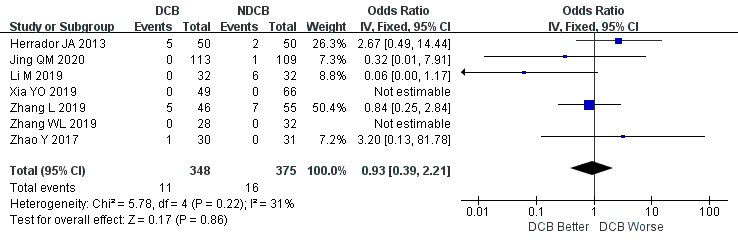
**
